# The Impact of Preoperative Hip Heterotopic Ossification Extent on Recurrence in Patients with Head and Spinal Cord Injury: A Case Control Study

**DOI:** 10.1371/journal.pone.0023129

**Published:** 2011-08-10

**Authors:** François Genêt, Claire Jourdan, Christine Lautridou, Clément Chehensse, Kambiz Minooee, Philippe Denormandie, Alexis Schnitzler

**Affiliations:** 1 Service de Médecine Physique et de Réadaptation, CHU R.Poincaré, Garches, France; 2 Service de chirurgie Orthopédique, CHU R.Poincaré, Garches, France; 3 Service de Rhumatologie et de Médecine Physique, CHU Erasme, Bruxelles, Belgique; Brigham and Women's Hospital, Harvard Medical School, United States of America

## Abstract

**Background:**

The preoperative Heterotopic Ossification (HO) extent is usually one of the main used criteria to predict the recurrence before excision. Brooker et al built a radiologic scale to assess this pre operative extent around the hip. The aim of this study is to investigate the relationship between the recurrence risk after hip HO excision in Traumatic Brain Injury (TBI) and Spinal Cord Injury (SCI) patients and the preoperative extent of HO.

**Methodology/Principal Findings:**

A case control study including TBI or SCI patients following surgery for troublesome hip HO with (case, n = 19) or without (control, n = 76) recurrence. Matching criteria were: sex, pathology (SCI or TBI) and age at the time of surgery (+/−4.5 years). For each etiology (TBI and SCI), the residual cognitive and functional status (Garland classification), the preoperative extent (Brooker status), the modified radiological and functional status (GCG-BD classification), HO localization, side, mean age at the CNS damage, mean delay for the first HO surgery, and for the case series, the mean operative delay for recurrence after the first surgical intervention were noted.

**Conclusions/Significance:**

The median delay for first HO surgery was 38.6 months (range 4.5 to 414.5;) for the case subgroup and 17.6 months (range 5.7 to 339.6) for the control group. No significant link was found between recurrence and operative delay (p = 0.51); the location around the joint (0.07); the Brooker (p = 0.52) or GCG-BD status (p = 0.79). Including all the matching factors, no significant relationship was found between the recurrence HO risk and the preoperative extent of troublesome hip HO using Brooker status (OR = 1.56(95% CI: 0.47–5.19)) or GCG-BD status (OR class 3 versus 2 = 0.67(95% CI: 0.11–4.24) and OR class 4 versus 2 = 0.79(95%CI: 0.09–6.91)). Until the pathophysiology of HO development is understood, it will be difficult to create tools which can predict HO recurrence.

## Introduction

Secondary orthopaedic complications after central nervous system lesion are common, mainly after Traumatic Brain Injury (TBI), but also after Spinal Cord Injury (SCI) and in a smaller proportion after stroke or cerebral anoxia [1]. Among them, Heterotopic Ossifications (HO) are frequent (from 11 to 76%) and induce pain and limit range of motion until complete ankylosis or vessel and nerve compression [1], [2], [3]. Nowadays, the predisposing factors to HO are well described for TBI and SCI patients: associated bone fracture, sepsis, prolonged immobilization, neurovegetative disorders, etc. [4], [5], [6], [7], [8], [9], [10], [11], [12].

The only effective treatment is still surgery for excision [1], [2], [3], [13]. Complications of these interventions are obviously hematoma, fractures and sepsis which depend on surgical and perioperative procedures. Indeed this patient group is complex, often with several co-morbid factors. Recurrence appears to depend not only on the surgical procedure but equally the etiology and the HO features [13], [14], [15], [16]. However, postoperative recurrence rate is rarely assessed for several reasons: there is no generally accepted definition (clinical or X-Ray diagnosis of recurrence); follow up is not standardised; surgical techniques change, mainly regarding peri operative and medical management. The delay of recurrence after surgery seems to be the same as that for the initial occurrence: within the first 3 months according to Gacon and Ippolito [17], [18] and between 3 and 6 weeks according to Stover [15]. Several studies have attempted to report the incidence of recurrence after HO excision [18], [19], [20], [21], [22], [23], [24], [25], [26], [27], [28], [29], [30], [31]. However, in most cases, these studies were descriptive (without statistical analysis) and involved a small sample of patients, frequently heterogeneous. The results of a meta analysis including 16 studies and 255 patients [32] suggested that there is no relationship between recurrence and operative delay, however, the average operative delay from neurological damage or HO diagnosis to surgery was widely dispersed in the studies included (13 to 30 months). The mean recurrence rate was estimated at 19.8% (IC 95%: 14,4–26,1%) but outcome measures were disparate (recurrence of decreased range of motion, ankylosis, X-Ray recurrence and surgical indications). In a previous study including a large sample of patients (357 patients, 539 surgeries) suffering surgery for troublesome HO after CNS lesion we found a mean recurrence rate of 5.8% (31 surgeries) [1].

**Figure 1:**
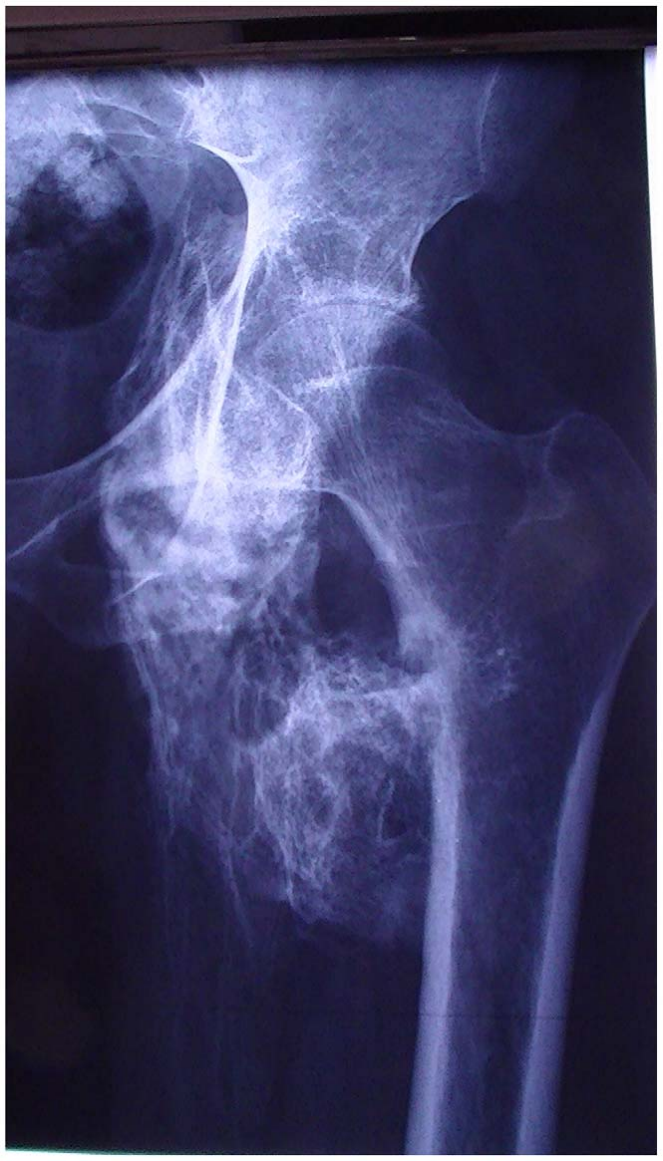
Hip Medial Heterotopic Ossification (Brooker Class IV).

**Figure 2:**
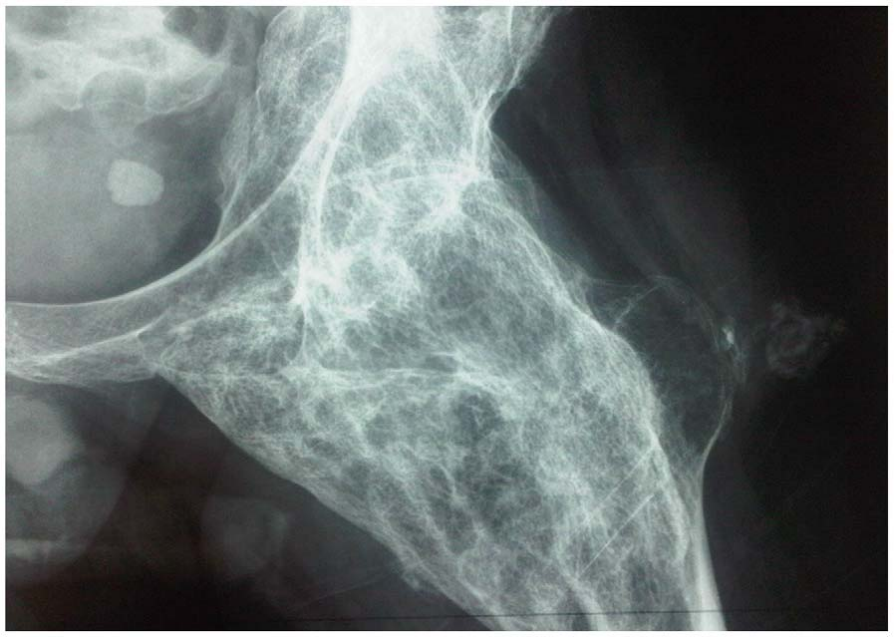
Encircling Hip Heterotopic Ossification.

Brooker et al., in 1973, proposed a method to classify the degree of ectopic bone formation about the hip following total hip arthroplasty [33]. They defined 4 classes (Class I: island of bone within the soft tissues about the hip, Class II: bone spurs from the pelvis or proximal end of the femur leaving at least one centimeter between opposing bone surfaces, Class III: bone spurs from the pelvis or proximal end of the femur, reducing the space between opposing bone surfaces to less than one centimeter, Class IV: apparent bone ankylosis of the hip, figures 1 and 2). Later, Stover et al. suggested that the extent of the initial HO is the most important factor in predicting postoperative recurrence in SCI patients [15]. Several teams have tried to find a link between the risk of recurrence and the pre operative extent of the HO however, their results were based on clinical observations and not a real scientific assessment [14]. The aim of this study was to assess if there is a relationship between the recurrence risk and the pre operative extent of hip HO in two large, homogenous samples of TBI and SCI patients undergoing surgery for troublesome HO in a single center and operated on by a single surgeon.

## Materials and Methods

### Ethics statement

The study was approved by the institutional review board, Comité de Protection des Personnes XI, Ile de France. It was a non-interventional study in which usual procedures were carried out and without any additional procedures (diagnosis or medical supervision). In France, patient consent is not needed for such an anonymous retrospective data analysis. We confirm that our named institutional review board specifically waived the need for consent for this study [Comité de Protection des Personnes Ile de France XI Pavillon Jacques Courtois - 2ème étage 20, rue Armagis 78105 Saint Germain en Laye Cedex. tél: 01.39.27.42.58 - fax: 01.39.27.49.01 mail: cppidf11@chi-poissy-st-germain.fr”].

### Study design

Case control study comparing clinical features, extent of hip HO and medical history of patients with troublesome HO requiring surgery after TBI or SCI with or without recurrence. Files of patients containing complete details of the pathology and HO were selected from a database of patients with central neurological system damage who had undergone surgery for troublesome HO in a single center specialized in this kind of treatment. Each patient in whom recurrence had occurred was matched with 4 control patients (no recurrence after 3 months of follow up) who were randomly selected by the manager of the Medical Information Department in files of patients with a follow up ensured in our physicians' network. Each TBI patient was matched with TBI controls and similarly for SCI patients. Matching criteria were: sex, pathology (TBI or SCI), and age at the time of surgery (+/−4.5 years). The residual cognitive and functional status (only for TBI patients, Garland classification [24]), the Brooker status [33], the modified radiological and functional GCG-BD classification of HO [14], the HO localization (around the joint), the side (right or left), the age at the time of CNS damage (months), the delay for the first HO surgery (months), the diagnosis delay for recurrence from the first surgery (surgeon consultation even if the diagnosis was certainly done before by the physicians who followed the patients after surgery)) (months) and the operative delay for recurrence from the first surgery (months) were recorded for each patient. The Brooker status was found in the surgical file. The GCG-BD was assessed retrospectively by another surgeon and a physician (Physical Medicine and Rehabilitation) who were blinded to the Brooker status, using preoperative X-Rays and reports.

### Constitution of the case database [1]

This single-centre study concerned a period between May 1993 and November 2009. Patients had been referred for a specialized neuro-orthopedic consultation for troublesome HO following CNS damage. Indications for surgery were loss of ROM with functional repercussions, ankylosed joint, and nerve or vessel compression. The surgical and immediate post-operative assessments were performed by the same surgeon.

Pre-operative investigations did not include serum alkaline phosphatase levels, Tc99 bone scanning or positivity for human leukocyte antigen B27 [32]. Several patients had received prophylactic treatment for HO, such as nonsteroidal anti-inflammatory drugs (for pain) or radiation therapy between 1993 and 2002 (this treatment was discontinued, probably due to lack of effectiveness).

A standardised surgical approach was used for each HO location. The surgical goal was resection of the amount of bone necessary to allow restoration of motion in all planes. Each patient underwent regular clinical examinations and x-rays; they were hospitalized in a surgical care unit for about 1 week, and then received regular consultations in rehabilitation units (inpatient care followed by outpatient care for a minimum of 1 year).

Patients were followed up in the rehabilitation unit of the same institution if they lived locally or by out-patient surgical consultation if they lived far away for about 3 months. For those patients, the prolonged supervision was organized by the physician of PMR over several months or years after these surgeries. The majority of these cases and controls are always followed by our colleagues. Therefore, all the troublesome recurrences had been diagnosed this especially as we work with a network agreement with these units: the surgeon would have been alerted.

### Statistical analysis

Statistical analysis was carried out with “R” Software® version 2.10.1 (Copyright (C) 2009. The R Foundation for Statistical Computing). Data are reported as numbers and percentages for categorical variables; median, minima, maxima and interquartile range for quantitative variables. Distributions of operative delays for first HO excision in the control and case samples were further described in a box plot.. Univariate tests used Kruskal-Wallis for quantitative variables, and Fisher tests for qualitative variables. All tests were two tailed, and a p value<0.05 was considered statistically significant. Correlation between Brooker and GCG-BD classification in the general sample of patients was assessed by Kendall's Tau.

The association between the Brooker or the GCG-BD status and the recurrence of HO was tested by a Fisher test.,Then two multivariate logistic models were carried out, to adjust for variables used for pairing The dependent variable was the presence of recurrence. Independent variables were gender and age, and either Brooker or GCG-BD(reference category was status 2 for GCG-BD). Adjusted odds ratios were calculated with 95% confidence intervals.

## Results

We found 19 cases of recurrence in our series of 539 surgeries since 1993 after TBI (9/19) and SCI (10/19). Following the matching criteria, we randomly selected 76 control files of TBI (36/76) and SCI (40/76) patients without recurrence after 3 months after surgery into the surgical database.

### Total population (Table 1)

**Table 1:** Demographic data and univariate analysis.

			Demographic data		Univariate analysis
			Case (n = 19)10 SCI9 TBI	Control (n = 76)40 SCI36 TBI	p
Sex ratio	Total		17 men (89.5%)	68 men (89.5%)	
	TBI		7 men (77.8%)	28 men (77.8%)	
	SCI		10 men (100.0%)	40 men (100.0%)	
Location	Hip		19 (100.0%)	76 (100.0%)	
Location around the joint	ANT		9 (47.4%)	34 (44.7%)	0.07
	POST		2 (10.5%)	12 (15.8%)	
	MEDIAL		5 (26.4%)	20 (26.3%)	
	LATERAL		0 (0.0%)	9 (11.8%)	
	ENCIRCLING		3 (15.8%)	1 (1.3%)	
Side (left/right)			7/12	32/44	0.75
Age at surgery median : IQR (min-max) (years)	Total		30.7 ; 25.8–39.1 (16.4–53.0)	30.0 ; 24.9–37.0 (15.0–56.0)	0.71
	TBI		29.8 ; 24.5–30.7 (16.4–38.6)	29.2 ; 23.7–31.2 (15.0–38.7)	0.84
	SCI		36.0 ; 28.4–42.5 (21.9–53.0)	35.7 ; 26.8–42.5 (18.5–56.0)	
Time from CNS damage to first surgery – median : IQR (min-max) (months)	Total		38.6; 9.5–63.6 (4.5–414.5)	17.6; 11.1–43.7 (5.7–339.6)	0.53
	TBI		10.0 ; 8.8–38.9 (5.6–50.1)	13.0 ; 9.3–26.7 (5.7–116.5)	0.50
	SCI		63.5 ; 32.5–202.2 (4.5–414.5)	24.5 ; 14.3–60.2 (7.2–339.6)	0.07
Brooker Status	Total	1	0/19 (0.0)	0/76 (0.0)	0.59
		2	0/19 (0.0)	0/76 (0.0)	
		3	11/19 (57.9)	50/76 (65.8)	
		4	8/19 (42.1)	26/76 (34.2)	
	TBI	1	0/9 (0.0)	0/36 (0.0)	1
		2	0/9 (0.0)	0/36 (0.0)	
		3	8/9 (88.9)	32/36 (88.9)	
		4	1/9 (11.1)	4/36 (11.1)	
	SCI	1	0/10 (0.0)	0/40 (0.0)	0.49
		2	0/10 (0.0)	0/40 (0.0)	
		3	3/10 (30.0)	18/40 (45.0)	
		4	7/10 (70.0)	22/40 (55.0)	
GCG-BD Status	Total	1	0/19 (0.0)	0/76 (0.0)	0.79
		2	2/19 (10.6)	6/76 (7.9)	
		3	10/19 (52.6)	44/76 (57.9)	
		4	7/19 (36.8)	26/76 (34.2)	
	TBI	1	0/9 (0.0)	0/36 (0.0)	0.71
		2	2/9 (22.2)	6/36 (16.7)	
		3	7/9 (77.8)	26/36 (72.2)	
		4	0/9 (0.0)	4/36 (11.1)	
	SCI	1	0/10 (0.0)	0/40 (0.0)	0.49
		2	0/10 (0.0)	0/40 (0.0)	
		3	3/10 (30.0)	18/40 (45.0)	
		4	7/10 (70.0)	22/40 (55.0)	
Garland StatusFor TBI	1		0/9 (0.0)	1/36 (2.8)	0.28
	2		2/9 (22.2)	5/36 (13.9)	
	3		1/9 (11.1)	1/36 (2.8)	
	4		1/9 (11.1)	15/36 (41.6)	
	5		5/9 (55.6)	14/36 (38.9)	

**Figure 3:**
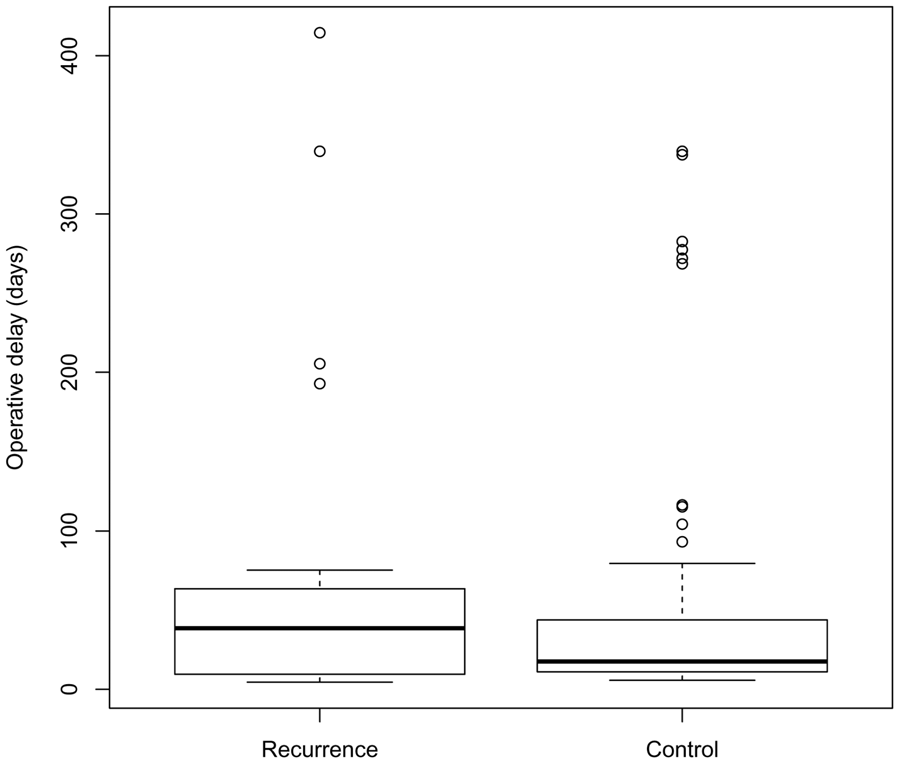
Operative delay for first HO excision in the case sample (‘recurrence”) and in the control sample. The upper and lower hinges indicate the 25^th^ and 75^th^ percentiles, the line in the box indicates the median.

95 patients with hip HO were included (85 men; SR: 81.3%). Median age was 30.2 (range 15.0 to 56.0, IQR 24.8; 37.7). Median operative delay for first HO excision was 17.9, (range 4.5 to 415.5; IQR: 10.9; 49.7, figure 3). Locations around the joint were anterior (43; 45.3%), posterior (14; 14.7%), medial (9; 9.5%), lateral (25; 26.3%) and encircling (4; 4.2%). The median follow-up by the surgeon was 10.3months (range 0.7 to 159.4; , IQR: 5.7; 30.0).

Based on Surgeon's files, the Brooker status on preoperative X-Ray was 61 (64.2%) patients with status 3 and 34 (35.8%) with status 4.

Based on preoperative X-Rays and functional abilities, the distribution of the GCG-BD status was class 2: 8 patients (8.4%), class 3: 54 patients (56.8%) and class 4: 33 patients (34.7%).

### Case series (Table 1)

There were 19 cases of recurrence (17 men, SR: 89.5%), 19 hips (7 left side). Median age at CNS damage was 30.7 years (range 16.4 to 53.0; IQR: 25.8–39.1). Median delay for the recurrence diagnosis from the first surgery by the surgeon was 2.57 months (range 1.1 to 62.4; IQR 1.6–3.9). Median delay for first HO surgery was 38.6 months (range 4.5 to 414.5; IQR 9.5–63.6) and the median follow-up by the surgeon was 20.1 months (range 2.5 to 95.3; IQR: 6.4–69.9).

### Control series (Table 1)

76 patients had no recurrence following surgical intervention for troublesome HO (68 men, SR: 89.5%), 76 hips (32 left side). Median age at CNS damage was 30.0 years (range 15.0 to 56.0; IQR: 24.9–37.0). The median delay for first HO surgery was 17.6 months (range 5.7 to 339.6; IQR: 11.1–43.7) and the median follow-up by the surgeon was 7.44 months (range 2.7 to 78.5; IQR: 5.6–27.7).

### Univariate analysis (Table 1)

No relationship was found between recurrence and operative delay (p = 0.51), HO side (p = 0.75), location around the joint (p = 0.07); Brooker status (p = 0.52) or GCG-BD status (p = 0.79).

A strong correlation was found between Brooker status and GCG-BD status (Kendall's Tau = 0.88, p<0.00001). When the TBI and SCI subgroups were dichotomized, no link between Brooker status and recurrence was found for either group (p = 0.74 for TBI; p = 0.48 for SCI), nor between GCG-BD status and recurrence (p = 0.71 for TBI; p = 0.49 for SCI) despite the significant difference in operative delay between these two subgroups.

### Logistic regression

When all the matching factors were taken into account, no significant relationship between Brooker status and HO recurrence risk was found (OR = 1.56 (95% CI: 0.47–5.19) nor between GCG-BD status and HO recurrence risk (OR for GCG-BD class 3 versus class 2 = 0.67 (95% CI: 0.11–4.24); OR for GCG-BD class 4 versus class 2 = 0.79 (95% CI: 0.09–6.91)).

### Comparability between the two groups: TBI versus SCI (Table 2)

**Table 2:** Comparability between the two groups: TBI versus SCI.

		TBI (%)	SCI (%)	p
Age at first surgery				p<0.01
Mean +/− SD		27.6+/−SD	35.5+/−SD	
Range		15.0 to 38.7	18.5 to 56.0	
Time from accident to first surgery				p<0.01
Mean +/− SD		24.2+/−SD	81.1+/−SD	
Range		5.5 to 114.6	4.4 to 407.5	
Location around the joint	ANTERIOR	14 (31.1)	29(58.0)	p = 0.03
	POSTERIOR	6 (13.3)	8 (16.0)	
	MEDIAL	16 (35.6)	9 (18.0)	
	LATERAL	7 (15.6)	2 (4.0)	
	ENCIRCLING	2 (4.4)	2 (4.0)	
Brooker status	3	40 (65.6)	21 (34.4)	p<0.01
	4	5 (14.7)	29 (85.3)	
GCG-BD	2	8 (100.0)	0 (0.0)	p<0.01
	3	33 (61.1)	21(38.9)	
	4	4 (12.1)	29 (87.9)	

There was a significant difference between the two groups for sex (p<0.01, no women in the SCI sub group), age at first surgery (p<0.01), operative delay from accident to first surgery (p<0.01), location around the joint (p = 0.03), Brooker status (p<0.01) and GCG-BD status (p<0.01).

## Discussion

### Main results

No significant relationship was found between the preoperative extent of hip HO and recurrence risk after surgical excision. The two subgroups, TBI and SCI, were significantly different in terms of sex, age at first surgery, operative delay, location around the joint and the both classifications of HO extent. A strong relationship was found between the scores of the two classifications (Brooker and GCG-BD).

### Preoperative HO extent and recurrence risk

Preoperative extent of HO has previously been suggested as being the main risk factor for recurrence after excision [14], [15], [33], [34]. Stover et al. followed 38 HO excisions of 18 patients with SCI [15]. The mean operative delay after the accident was about 26 months (range: 11 months to 17 years). They found 13 recurrences with severe limitation of range of motion and all had a Brooker status above 3. Ebinger et al. found a significant link between the Glasgow coma scale score and the Brooker status for the hip HO (r = −0.83) [13]. They carried out a prospective study of 64 patients with HO (3 groups, I = severe TBI and local hip trauma (25 patients); II: isolated head injury (18 patients) and III: isolated local hip trauma (21 patients)) and their results revealed a tendency for a higher Brooker status score for group I than respectively for classes II and III. However, they did not find a difference for the Brooker status of the HO recurrence between the 3 classes at one and five years of follow up. They did not assess if the Brooker status was a predictive factor for recurrence. Garland et al. found a significant link between HO volume and maturity and recurrence risk after SCI (24 patients, 31 hips) [34]. However, they did not use the Brooker status, the severity of preoperative HO was assessed by a subjective X-Ray grading system: 1 = minimal, 2 = mild, 3 = moderate, 4 = severe, 5 = ankylosed. For TBI patients, they found a link between the HO score for a single patient (≥3) and the recurrence risk [24].

Seegenschmiedt et al. in a review [14], evoked some limitations of the Brooker classification. Firstly, the x-Ray incidence is estimated only in the frontal plane which could lead to underestimation of anterior and posterior HO development by superimposition, notably with the femor (Figure 1). Moreover, we can add that in our sample of patients (troublesome HO with surgical indications), the majority of patients had a status of III or IV which corresponds to voluminous HOs and a three-dimensional analysis seems to be more precise (Figure 1 and 2). Patients in class I are rarely symptomatic. The other limit, also highlighted by Seegenschmiedt et al., is that this classification cannot be transferred to other joints [14]. Since HO after CNS damage develops around joints of different sizes (hip, knee, shoulder elbow), the transposition of an assessment using the extent of HO in centimeters is difficult. This is why Hastings and Graham proposed a radiological and functional classification of HO about the elbow and forearm joint [14]. There were three classes, from non-symptomatic (Class 1) to ankylosis (Class 3). The second class is interesting because it includes range of motion decrease in different planes (sub class A: flexion/extension for the elbow and sub class B: pronation/supination for the forearm and Sub class C for both). This classification was not assessed by means of a clinical study. The German patterns-of-care study (PCS) proposed a general classification system (named GCG-BD) for HO development which could be used for any joint and based on X-Ray or CT scan. Their classification is based on the Brooker status with the addition of functional components (no, minor, major or complete functional deficit or symptoms of the involved joint or body segment).

The results of the present study found that Brooker and GCG-BD statuses were significantly higher for SCI than TBI patients. This is likely to be related to operative delay [1]. Because surgery is often proposed for TBI patients earlier than SCI patients, functional outcomes improve sooner since they have more ability to stand or walk. Furthermore, SCI patients frequently have a loss of sensation which can lead to a longer delay for diagnosis because of the absence of pain.

Even if the Brooker and GCG-BD classifications do not seem to be good markers for the recurrence risk, it would be interesting to assess if these classifications can predict other post operative complications such as fractures, hematoma or sepsis.

### Correlations between the two classifications

The results of this study showed a strong, significant correlation (k = 0.81) between the two classifications of preoperative HO extent, even if they were obtained by different means. This is probably due to the fact that most patients had quite severe neurological sequelae. Secondly, the score for pre operative HO extent was quite similar for all patients (III or IV). This is probably because Seegenschmiedt et al. were inspirited by the Brooker classification for the radiologic extent when they created their scale [14]. It seems likely that the degree of neurological sequelae has a smaller impact on Brooker status than the pre operative extent of HO. Moreover, functional ability depends on many factors such as the severity of cognitive and motor deficits, behavioral modifications, co-morbid factors (neurological, orthopaedic…) and pre injury status. This could make this classification insensitive for the assessment of risk factors for HO development or recurrence. Until the pathophysiology of HO development is understood, it will be difficult to create tools which can predict HO recurrence.

### Limits of the study

This study may lack power especially for the separate analysis of each etiology (TBI and SCI) because of the small size of each sample. However, to our knowledge no other study has assessed the impact of preoperative extent of HO on recurrence risk, particularly in such a large group of patients. The lack of comparability between the two groups (TBI and SCI) has previously been found [1]. The functional prognosis of gait recovery and the presence of sensory deficits are two of the major explanations for these differences. The matching strategy which respected the same etiology between case and control patients tends to reduce the impact of this limit. Finally, although inclusion of controls was random in our series, the sample only included the two more extended Brooker classes (3 and 4) which can entail a selection bias. This could be related to the specific recruitement in our institution of patients who are most bothered by their HO, as Brooker classes 1 or 2 POA require less often surgical treatment.

### Conclusion

No relationship was found between the preoperative extent of the troublesome hip HO and recurrence risk for patients with TBI or SCI. Surgery should be undertaken as soon as the co-morbid factors are under control.

## References

[1] Genet F, Jourdan C, Schnitzler A, Lautridou C, Guillemot D (2011). Troublesome heterotopic ossification after central nervous system damage: a survey of 570 surgeries.. PLoS One.

[2] Cipriano CA, Pill SG, Keenan MA (2009). Heterotopic ossification following traumatic brain injury and spinal cord injury.. J Am Acad Orthop Surg.

[3] Vanden Bossche L, Vanderstraeten G (2005). Heterotopic ossification: a review.. J Rehabil Med.

[4] Garland DE, Alday B, Venos KG (1984). Heterotopic ossification and HLA antigens.. Arch Phys Med Rehabil.

[5] Garland DE, Blum CE, Waters RL (1980). Periarticular heterotopic ossification in head-injured adults. Incidence and location.. J Bone Joint Surg Am.

[6] Hajek VE (1987). Heterotopic ossification in hemiplegia following stroke.. Arch Phys Med Rehabil.

[7] Minaire P, Betuel H, Girard R, Pilonchery G (1980). Neurologic injuries, paraosteoarthropathies, and human leukocyte antigens.. Arch Phys Med Rehabil.

[8] Rosin AJ (1975). Ectopic calcification around joints of paralysed limbs in hemiplegia, diffuse brain damage, and other neurological diseases.. Ann Rheum Dis.

[9] Sazbon L, Najenson T, Tartakovsky M, Becker E, Grosswasser Z (1981). Widespread periarticular new-bone formation in long-term comatose patients.. J Bone Joint Surg Br.

[10] Simonsen LL, Sonne-Holm S, Krasheninnikoff M, Engberg AW (2007). Symptomatic heterotopic ossification after very severe traumatic brain injury in 114 patients: incidence and risk factors.. Injury.

[11] Tsur A, Sazbon L, Lotem M (1996). Relationship between muscular tone, movement and periarticular new bone formation in postcoma-unaware (PC-U) patients.. Brain Inj.

[12] Hendricks HT, Heeren AH, Vos PE (2010). Dysautonomia after severe traumatic brain injury.. Eur J Neurol.

[13] Ebinger T, Roesch M, Kiefer H, Kinzl L, Schulte M (2000). Influence of etiology in heterotopic bone formation of the hip.. J Trauma.

[14] Seegenschmiedt MM, O. Heyd R (2008). Heterotopic ossifications: general survey for all sites... Medical Radiology, Radiotherapy for Non-Malignant Disorders.

[15] Stover SL, Niemann KM, Tulloss JR (1991). Experience with surgical resection of heterotopic bone in spinal cord injury patients.. Clin Orthop Relat Res.

[16] Chalidis B, Stengel D, Giannoudis PV (2007). Early excision and late excision of heterotopic ossifications after traumatic brain injury are equivalent: a systematic review of the literature.. J Neurotrauma.

[17] Gacon G, Deidier C, Rhenter JL, Minaire P (1978). [Ectopic bone formation in neurological lesions (author's transl)].. Rev Chir Orthop Reparatrice Appar Mot.

[18] Ippolito E, Formisano R, Caterini R, Farsetti P, Penta F (1999). Operative treatment of heterotopic hip ossification in patients with coma after brain injury.. Clin Orthop Relat Res.

[19] Carlier RY, Safa DM, Parva P, Mompoint D, Judet T (2005). Ankylosing neurogenic myositis ossificans of the hip. An enhanced volumetric CT study.. J Bone Joint Surg Br.

[20] Charnley G, Judet T, Garreau de Loubresse C, Mollaret O (1996). Excision of heterotopic ossification around the knee following brain injury.. Injury.

[21] Denormandie P, Viguie G, Denys P, Dizien O, Carlier R (1999). Results of excision of heterotopic new bone around the elbow in patients with head injuries. A series of 25 cases.. Chir Main.

[22] Frischhut B, Stockhammer G, Saltuari L, Kadletz R, Bramanti P (1993). Early removal of periarticular ossifications in patients with head injury.. Acta Neurol (Napoli).

[23] Fuller DA, Mark A, Keenan MA (2005). Excision of heterotopic ossification from the knee: a functional outcome study.. Clin Orthop Relat Res.

[24] Garland DE, Hanscom DA, Keenan MA, Smith C, Moore T (1985). Resection of heterotopic ossification in the adult with head trauma.. J Bone Joint Surg Am.

[25] Ippolito E, Formisano R, Caterini R, Farsetti P, Penta F (1999). Resection of elbow ossification and continuous passive motion in postcomatose patients.. J Hand Surg Am.

[26] Lazarus M, Guttmann D, Rich C, Keenan M (1999). Heterotopic ossification resection about the elbow.. Neurorehabilitation.

[27] McAuliffe JA, Wolfson AH (1997). Early excision of heterotopic ossification about the elbow followed by radiation therapy.. J Bone Joint Surg Am.

[28] Moore TJ (1993). Functional outcome following surgical excision of heterotopic ossification in patients with traumatic brain injury.. J Orthop Trauma.

[29] Roberts JB, Pankratz DG (1979). The surgical treatment of heterotopic ossification at the elbow following long-term coma.. J Bone Joint Surg Am.

[30] Sarafis KA, Karatzas GD, Yotis CL (1999). Ankylosed hips caused by heterotopic ossification after traumatic brain injury: a difficult problem.. J Trauma.

[31] Sorriaux G, Denormandie P, Martin JN, Kiefer C, Judet T (2005). [Excision of heterotopic new bone around the elbow in patients with head injury: 51 cases].. Rev Chir Orthop Reparatrice Appar Mot.

[32] Chalidis B, Stengel D, Giannoudis PV (2007). Early excision and late excision of heterotopic ossification after traumatic brain injury are equivalent: a systematic review of the literature.. J Neurotrauma.

[33] Brooker AF, Bowerman JW, Robinson RA, Riley LH (1973). Ectopic ossification following total hip replacement. Incidence and a method of classification.. J Bone Joint Surg Am.

[34] Garland DE, Orwin JF (1989). Resection of heterotopic ossification in patients with spinal cord injuries.. Clin Orthop Relat Res.

